# The Correlation Between Microbial and Physicochemical Properties of Beef Thigh Meat and Particle Size, Polydispersity Index, and Zeta Potential After Treating With Different Concentrations of Sodium Alginate Nanoparticles Containing Lemon Verbena Plant Leaf Essential Oil

**DOI:** 10.1002/fsn3.71126

**Published:** 2025-10-29

**Authors:** Mehran Sayadi, Elahe Abedi, Zolaikha Shiravani, Niayesh Ramyar, Issa Mohammadpourfard, Seyed Mohammad Bagher Hashemi, Zahra Eskandari, Narjes Jamali

**Affiliations:** ^1^ Department of Food Safety and Hygiene School of Health, Fasa University of Medical Sciences Fasa Iran; ^2^ Department of Food Science and Technology Faculty of Agriculture, Fasa University Fasa Iran; ^3^ Department of Food Hygiene Faculty of Veterinary Medicine, Amol University of Special Modern Technologies (AUSMT) Amol Iran; ^4^ Student Research Committee Fasa University of Medical Sciences Fasa Iran

**Keywords:** beef thigh, *lemon verbena* essential oil, nanoparticles, shelf life, sodium alginate

## Abstract

In the present study, the effect of sodium alginate nanoparticles (SANPs) combined with *lemon verbena* plant leaf essential oil (LVEOs) (concentrations of 0.25%, 0.5%, and 1% w/v) as an edible coating on the chemical and microbial changes of beef thigh meat during refrigerated storage was investigated. The dynamic light scattering (DLS) and zeta sizer analyses of SANPs incorporating LVEOs yielded the following parameters across LVEO concentrations: at 1%, the hydrodynamic diameter (Z‐average) was 32.3 nm with a polydispersity index (PDI) of 0.681 and a zeta potential (ZP) of −39.9 mV; at 0.5%, the Z‐average was 88.6 nm with PDI 0.544 and ZP −30.1 mV; at 0.25%, the Z‐average was 207.7 nm with PDI 0.465 and ZP −21.7 mV. The data indicate an increasing particle size with decreasing LVEO concentration, with all PDIs below 0.7 and progressively less negative surface charge at lower LVEO content, suggesting reduced electrostatic stabilization at lower concentrations. According to scanning electron microscopy (SEM), the nanoparticles displayed a spherical morphology with smooth surfaces. The current investigation attributes spectral variations within 2800–3000 cm^−1^ to C—H modes associated with monoterpenes, whereas the 1000–1100 cm^−1^ bands reflect interactions between the C—O—C moieties of the polysaccharide and compounds present in the essential oil. With increasing essential oil concentrations (0.25%, 0.5%, and 1%), the intensities of several bands—particularly those in the —OH and C—H regions—became more pronounced. Thigh meat coated with SANPs + LVEOs (1% w/v) had the lowest microbial growth, 7.66 ± 0.06 log CFU/g for aerobic mesophilic (by 5.2‐log reduction compared with control), 6.44 ± 0.07 log CFU/g for coliforms (3.65‐log reduction), 6.76 ± 0.30 log CFU/g for lactic acid bacteria (3.67‐log reduction), and this treatment also had the lowest degree of lipid oxidation (thiobarbituric acid reactive substances or TBARS value) by 69.5% on Day 20 of storage. It also had the highest sensory score in overall acceptability. These results indicate that SANPs + LVEOs (1% w/v) can be used as an edible coating to enhance the shelf life of meat.

## Graphical abstract

Active sodium alginate coating incorporated with sodium alginate nanoparticles (SANPs) and lemon verbena essential oil (LVEOs) was prepared. The beef thigh coated with SANPs containing 2% LVEOs had better chemical and microbial quality than the control group. The beef thigh coated with SANPs containing 2% LVEOs showed the highest sensory scores after 20 days.

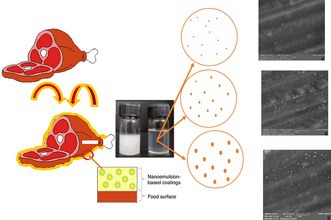

## 1 Introduction

During the storage period of meat, the two main factors that reduce the quality and make meat unusable are the growth of microorganisms, which cause microbial spoilage of meat, and the oxidation of lipids and proteins, which affect its sensory characteristics and nutritional value (Abedi, Tavakoli, et al. 2023; Hashemi et al. 2023; Ornaghi et al. 2024).

Today, researchers are exploring innovative methods to extend the shelf life of perishable foods while maintaining their quality and safety. Sodium alginate (SA), a biopolymer derived from seaweed, is widely used in the food industry due to its gelling and encapsulation properties. SA can create a protective barrier around the EO, preventing its premature release and ensuring a controlled release over an extended period. The mechanism for controlling release not only helps preserve food products but also enhances overall sensory acceptance (Kulig et al. 2017; Lourenço et al. 2020). Sodium alginate nanoparticles (SANPs) are biocompatible, biodegradable, and nontoxic nanoparticles that have been widely studied for their potential applications in food preservation (Yang et al. 2022). These nanoparticles have excellent film‐forming properties, making them ideal for creating a protective coating on food surfaces. SANPs containing essential oils (EOs) derived from plants can be produced to coat meat products (Mousavi Tarsi et al. 2021; Senturk Parreidt et al. 2018). Applying SANPs containing EOs for coating meats offers several advantages in terms of long‐term preservation. Firstly, the antimicrobial properties of EOs can help inhibit the growth of pathogenic bacteria, reducing the risk of foodborne illnesses. Additionally, the antioxidant properties of both the EO and the NPs can delay lipid oxidation, thereby maintaining the sensory features of the meat, including its color, flavor, and texture, and maintaining the freshness of the meat for an extended period (Alexandre et al. 2021; Lu et al. 2021; Shahbazi and Shavisi 2019; Zhang et al. 2022).

EOs have long been recognized for their antimicrobial properties, making them a natural choice for enhancing the shelf life of perishable food items (Ju et al. 2019). *Lemon verbena* plant leaf essential oil (LVEOs) is known for its pleasant citrusy aroma and potent antibacterial and antioxidant properties, and the major constituents of this essential oil are geranial, neral, spathulenol, and limonene, along with phenolic compounds such as transferulic acid, hesperidin, and ρ‐coumaric acid (Hematian Sourki et al. 2021). Nanoencapsulation of this oil in sodium alginate particles enhances its functionality through controlled release and improved antimicrobial activity. Encapsulation protects volatile and sensitive compounds (particularly geranial and neral), enabling their gradual release and maintaining effective concentrations on the meat surface during storage (Abedi, Akhavan, et al. 2023; Gholamhossein Tabar et al. 2025). The nanoscale system also increases the interaction of limonene with microbial cell membranes, thereby enhancing antibacterial efficacy via disruption of membrane integrity, leakage of intracellular contents, interference with membrane proteins, and inhibition of cellular respiration (Gutiérrez‐Pacheco et al. 2023; Liao et al. 2023). In addition, the alginate coating provides a physical barrier that limits oxygen diffusion and moisture loss, thereby enhancing the antioxidant effects of phenolics (e.g., hesperidin and transferulic acid), reducing lipid oxidation, and ultimately improving the chemical and sensory quality of beef (Lourenço et al. 2020).

Previous studies have shown that this essential oil has been able to increase the shelf life of fish meat effectively (Rezaeifar et al. 2020), turkey meat (Sayadi et al. 2024), and chicken breast (Hosseini et al. 2021). Accordingly, this study aimed to use SANPs containing different concentrations (0.25%, 0.5%, and 1%) of LVEOs as a promising approach for the long‐term preservation of beef thighs. In this regard, the relationship between EO concentrations and particle size, polydispersity index (PDI), zeta potential (ZP), morphology, and Fourier transform infrared spectroscopy (FTIR) was examined. The SANPs containing different concentrations (0.25%, 0.5%, and 1%) of LVEOs were then evaluated for their effectiveness on pH, TVB‐N, TBARS, microbial count, and sensorial attributes of beef thigh meat. The objective of this question was to investigate the correlation among EO concentrations, particle size, PDI, and ZP with respect to the long‐term preservation of beef thighs.

## 2 Materials and Methods

### 2.1 Materials

*Lemon verbena* EO was purchased from Aminsan Company (Tehran, Iran). Tween 80, sodium alginate, trichloroacetic acid, hydrochloric acid (HCL), sulfuric acid (H_2_SO_4_), 2‐thiobarbituric acid, potassium iodide, sodium thiosulfate, and other chemical compounds were provided by Sigma Aldrich Company (St. Louis, USA). Bacterial cultures included Violet Red Bile Agar (VRBA), plate count agar (PCA), and de Man, Rogosa, and Sharpe agar (MRS), which were supplied by the Merck Company (Darmstadt, Germany).

### 2.2 Preparation of Sodium Alginate Solution Containing *L. Verbena* EOs

Nanoparticles were prepared by the ionic gelation method (Thomas et al. 2020). Different EO concentrations (0.25%, 0.5%, and 1% w/v) were dissolved in a sodium alginate aqueous solution (0.5% w/v) with Tween 80. Next, an aqueous calcium chloride solution (0.1% w/v) was used as a crosslinker to form SANPs. Dynamic light scattering (DLS) was used to determine the size of the prepared nanoparticles.

### 2.3 Beef Thigh Coating

Sterile beef thigh samples were immersed (5 min) in SANPs solution containing *L. verbena* EOs and dried in the air for 3 min before packaging. Then, the samples were packed in plastic bags and stored in the refrigerator for 20 days. On Days 0, 5, 10, 15, and 20, the samples were analyzed for microbial, chemical, and sensory properties.

Different treatments were prepared and evaluated as follows:

A: Control treatment (control).

B: Sample coated with sodium alginate coating (0.5% w/v) (SA).

C: Sample coated with SANPs coating and 0.25% *L. verbena* EOs (SA + 0.25% LVEOs).

D: Sample coated with SANPs coating containing 0.5% *L. verbena* EOs (SANPs+0.5% LVEOs).

E: Sample coated with SANPs coating containing 1% *L. verbena* EOs (SANPs+1% LVEOs).

### 2.4 Determination of Particle Size Distribution and Zeta Potential

The particle size of NEs was determined using a laser diffraction system equipped with a 4 mW He‐Ne laser operating at 633 nm. Measurements were performed with a viscosity setting of 8.76 mPa.s at 25°C, using detection angles of 70° and 90° on the Nano ZS analyzer (Malvern Instruments Ltd., UK). Data processing, including hydrodynamic size, z‐average, and polydispersity index, was conducted with DTS software version 5.02 (Karimi‐Khorrami et al. 2022).

### 2.5 Morphological Properties

#### 2.5.1 Scanning Electron Microscopy (SEM)

A 0.5% sample was diluted 1000‐fold with distilled water, and a drop of the suspension was deposited onto a glass lamella. After drying at ambient temperature, the specimen was sputter‐coated with gold and subsequently imaged using a field emission scanning electron microscope (FE‐SEM, WEGA3 SB, TESCAN, Czech Republic) operated at 20 kV with a magnification of 35,000× (Amiri et al. 2024).

#### 2.5.2 Fourier Transform Infrared (FTIR) Spectroscopy

FTIR spectroscopy was performed to analyze functional groups using an FTIR spectrophotometer (Tensor II, Bruker, Germany) in the wavelength range of 4000–500 cm^−1^.

### 2.6 Chemical Analysis

#### 2.6.1 pH Measurement

The samples (10 g) were mixed with distilled water (90 mL) for 2 min, and then, to measure the pH of the samples, a digital pH meter (Zag‐ChemiCo., Tehran, Iran) was used (Hassanzadeh et al. 2018).

#### 2.6.2 Thiobarbituric Acid Reactive Substances (TBARS)

To measure the TBARS test, 10 g of the crushed sample was mixed well with 35 mL of trichloroacetic acid (5%) and 1 mL of butylated hydroxytoluene (0.5%) and then filtered. 5 mL of the filtered material was mixed with 5 mL of 2‐thiobarbituric acid (0.02 M) and placed in a water bath at 100°C for 1 h and finally cooled to room temperature. The pink solution formed at 532°C was read spectrophotometrically against a blank. TBARS is expressed based on milligrams of malondialdehyde per 1 kg of sample (mg MDA/kg sample), according to the following equation:

(1) $$\text{TBARS values}=50\times \frac{A-B}{w}$$

Here, *A* and *B* are the absorbance of the sample and control solution, receptivity, and w is the weight of the beef thigh sample (Moosavi‐Nasab et al. 2018).

#### 2.6.3 Total Volatile Basic Nitrogen (TVB‐N)

The determination of TVB‐N was performed via the Kjeldahl distillation method. 10 g of the sample was placed in a distillation flask, 2 g of magnesium oxide was added, and 300 mL of distilled water was added. The distillation flask was connected to the distillation apparatus. An Erlenmeyer containing 25 mL of 2% boric acid and a few drops of methyl red reagent was also placed at the end of the condenser. Since the distillation started and continued for 15 min, the contents reached almost 50 mL. Finally, 0.1 N sulfuric acid was used for titration. TVB‐N is expressed based on milligrams of nitrogen per 100 g of sample, according to the equation below:

(2) $$\mathrm{TVB}-N=\frac{\left(V_{1}-V_{2}\right)\times N\times 100\times 14\times 50}{m\times 5}$$

Here, *V*_1_ and *V*_2_ are mL of H_2_SO_4_ used for the sample and the blank, respectively. *N* is the normality of H_2_SO_4_, and m is the weight of the beef thigh sample (g) (Amiri et al. 2024).

### 2.7 Microbial Analysis

In sterile conditions on different days (0, 5, 10, 15, and 20), 10 g of meat samples were transferred into individual stomaching bags containing 90 mL of sterile peptone water solution (0.1%) and homogenized in a stomacher (Stomacher 400, Circulator, Seward) for 2 min. Serial decimal dilutions were prepared by diluting 1 mL of homogenate in 9 mL of peptone water; the culture of surface method, 0.1 mL from appropriate dilutions was spread on the surface of specific media as follows: for the growth of bacteria, aerobic mesophilic bacteria using plate count agar, incubation time 48 h at 37°C; coliforms using violet red bile agar, incubation period 24 h at 37°C, and Lactic acid bacteria (LAB) using MRS agar after 24 h of incubation at 35°C were counted (Shafiei et al. 2024).

### 2.8 Sensory Evaluation

Ten semi‐trained people evaluated the sensory characteristics (odor, texture, color, and overall acceptance) of all the treatments using the 9‐point hedonic method during different periods of refrigerated storage. Where 1 = extremely bad and 9 = extremely good, a score of 5 was established as the threshold for minimum acceptability.

### 2.9 Statistical Analysis

Statistical analysis of the data was performed using one‐way analysis of variance (ANOVA) and the Statistical Package for the Social Sciences (SPSS) version 26. The comparison of means was performed with Tukey's test, and a *p* < 0.05 value was considered significant. All tests were analyzed in triplicate.

## 3 Results and Discussion

### 3.1 SEM Analysis, Particle Size, Polydispersity Index, and Zeta Potential

**FIGURE 1:**
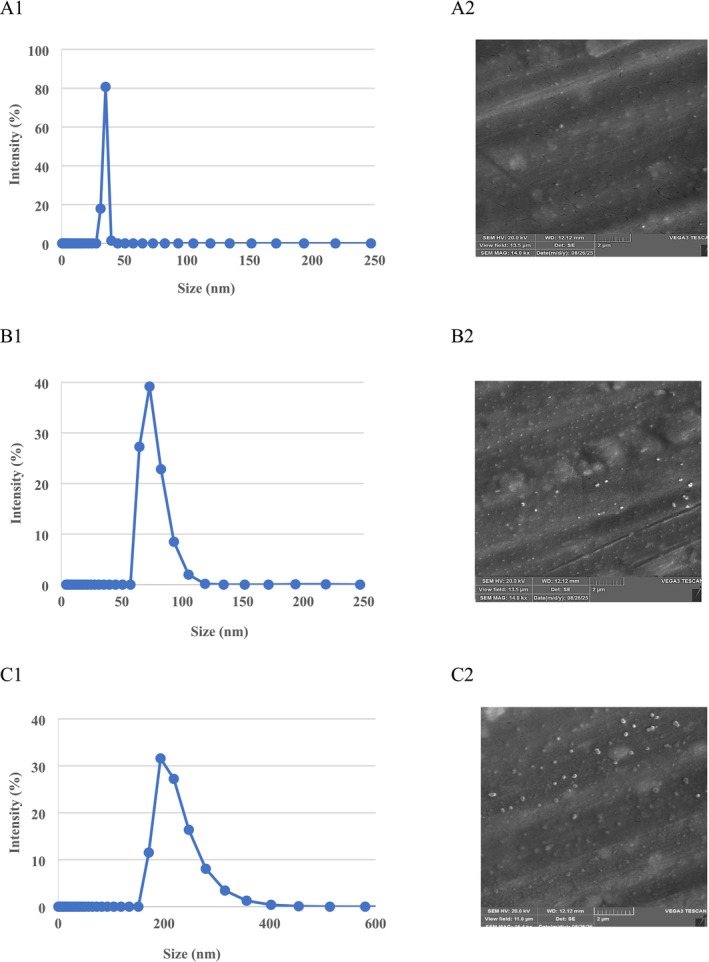
The particle size (DLS) and scanning electron microscopy (SEM) of the sodium alginate nanoparticles (SANPs) containing *lemon verbena* plant leaf essential oil at concentrations of 1% (A1 and A2), 0.5% (B1 and B2), and 0.25% (C1 and C2).

**FIGURE 2:**
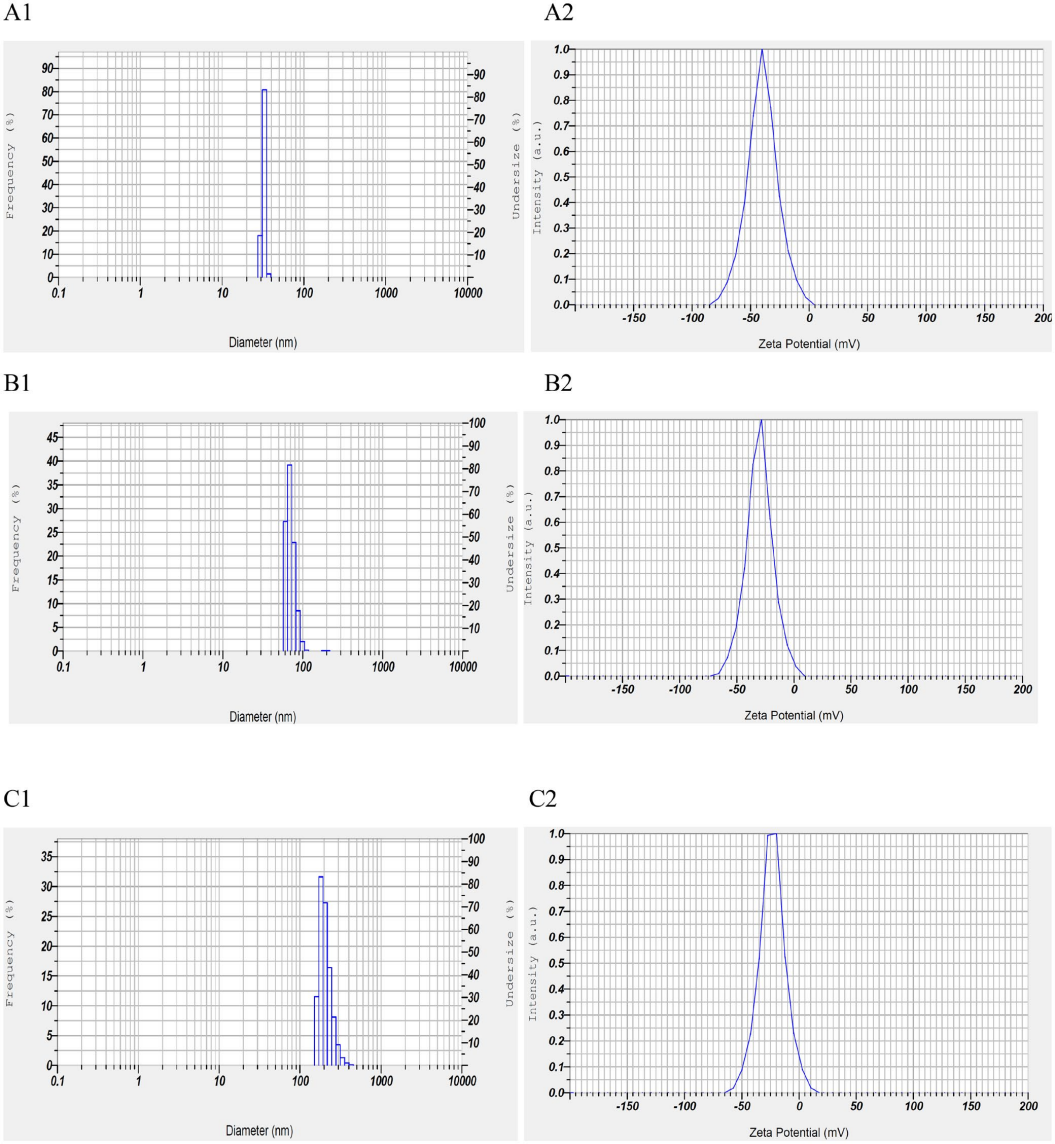
The poly dispersity index (PDI) and zeta size of the sodium alginate nanoparticles (SANPs) containing *lemon verbena* plant leaf essential oil (LVEOs) at concentrations 1% (A1 and A2), 0.5% (B1 and B2), and 0.25% (C1 and C2).

The size of the nanoparticle coating solution was 207.7 nm (0.25%), 88.6 nm (0.5%), and 32.3 nm (1%) (Table 1, Figure 1), which was in agreement with Osanloo et al. (2023) who reported that the particle size of the SANPs with EOs was 195 nm. In another study, Baek et al. (2021) claimed that the mean particle size of nanoparticles incorporating SANPs and grapefruit seed extract was 206.8 nm, which is more than the present results. The corresponding PDIs (Table 1, Figure 2) of SANPs containing LVEOs at concentrations of 0.25%, 0.5%, and 1% were 0.465, 0.544, and 0.681, while ZP values (Table 1, Figure 2) were determined as −21.7, −30.1, and −39.9 mV, respectively. Since ZP reflects the overall surface charge of lipid vesicles, it is considered a fundamental parameter in assessing nanocarrier stability (Talesh et al. 2025). Stronger negative or positive ZP values suggest greater electrostatic repulsion, reducing the likelihood of particle aggregation (Amiri et al. 2024; Huang et al. 2020; Malvano et al. 2022; Wang et al. 2021). In contrast, ZP values close to neutrality (−20 to +20 mV) indicate limited stability due to insufficient electrostatic interactions (Bhattacharjee 2016). The relatively higher negative ZP of the NEs implies enhanced physical stability (Laein et al. 2024; Safaeian Laein et al. 2023). In this regard, the higher concentration (1%) showed a higher ZP (−39.9 mV), followed by 0.5% (−30.1 mV) and 0.25% (−21.7 mV). In lipid‐based nanocarrier systems, particle size distribution and PDI represent vital physical attributes that must be considered in both food and pharmaceutical formulations. These factors directly affect properties such as stability, processability, product performance, and appearance. To ensure consistent quality, it is crucial to perform reproducible analyses of mean size, polydispersity, and charge (Danaei et al. 2018). Moreover, they are considered a key determinant of lipid nanocarrier performance, influencing stability, encapsulation efficiency, drug release kinetics, biodistribution, and cellular uptake. For particle size distribution assessment, the commonly used parameter is the PDI. Values below 0.05 are typically observed only in highly monodisperse standards. In contrast, values above 0.7 reflect very broad particle size distributions, rendering the sample unsuitable for analysis by DLS. Most size distribution algorithms operate effectively within the intermediate PDI range of 0.05–0.7 (Danaei et al. 2018). PDI values range between 0.0 (monodisperse) and 1.0 (highly polydisperse). For polymeric nanoparticles, values ≤ 0.2 are acceptable, whereas lipid‐based carriers, including liposomes and nanoliposomes, are considered suitably homogeneous at PDIs ≤ 0.3.

**TABLE 1:** The size, PDI, and zeta potential of the sodium alginate nanoparticles (SANPs) containing *lemon verbena* plant leaf essential oil (LVEOs) at concentration.

Histogram operations	Nanoemulsion		
	1%	0.5%	0.25%
Size (median)	32.6 nm	74.0 nm	199.5 nm
Mode	32.6 nm	70.1 nm	187.6 nm
% Cumulative (2)	10.0 (%)—29.4 (nm)	10.0 (%)—61.9 (nm)	10.0 (%)—168.5 (nm)
% Cumulative (6)	50.0 (%)—32.6 (nm)	50.0 (%)—74.0 (nm)	50.0 (%)—199.5 (nm)
% Cumulative (10)	90.0 (%)—34.6 (nm)	90.0 (%)—100.2 (nm)	90.0 (%)—259.5 (nm)
Mean	32.3 nm	88.6 nm	207.7 nm
Span	0.159778	0.518018	0.456031
PDI	0.681	0.544	0.465
Zeta Potential (Mean)	−39.9 mV	−30.1 mV	−21.7 mV

The SEM image of SANPs containing different concentrations of LVEOs is shown in Figure 1. The analysis revealed that the nanoparticles exhibited a spherical shape with smooth surfaces. The particle size in a 1% LVEO concentration was significantly smaller than that in 0.5% and 0.25% concentrations, confirming the data obtained by DLS. This structural characteristic is considered advantageous for the homogeneous distribution of essential oils as follows: 1% > 0.5% > 0.25% in food matrices, consistent with the findings of Khorrami et al. (2021), Amiri et al. (2024) and Akhavan et al. (2021).

### 3.2 FTIR Results

**FIGURE 3:**
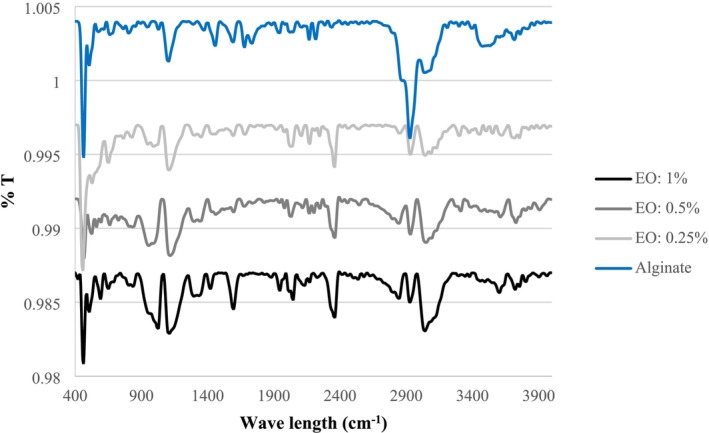
FTIR spectra of the sodium alginate nanoparticles (SANPs) containing *lemon verbena* plant leaf essential oil (LVEOs) at concentrations of 1%, 0.5%, and 0.25%.

The FTIR technique was employed to identify the interactions between SA and LVEOs at different concentrations (0.25%, 0.5%, and 1%) (Figure 3). The results confirmed the physical interactions between alginate and LVEOs compounds. The spectrum of SA exhibited several prominent peaks in the regions of 3200–3400 cm^−1^ and around 2900 cm^−1^, corresponding to the stretching vibrations of —OH and C—H groups, respectively (Lopes et al. 2025; Mahcene et al. 2020). The asymmetric stretching vibrations at around 1590–1610 cm^−1^ and the symmetric stretching at around 1400–1420 cm^−1^ were assigned to the carboxylate groups (—COO), while the absorption at 1020–1080 cm^−1^ confirmed the presence of the C—O—C bond (Kowalonek et al. 2023; Mahcene et al. 2020), which indicates the basic structure of alginate (Bhatia et al. 2023). Since the main components of LVEOs include limonene, citral, geranial, and nerol, noticeable changes were observed in the FTIR spectra of the samples containing essential oil at different concentrations. The intensity variation and slight shifts in the –OH stretching band (3200–3400 cm^−1^) confirmed the hydrogen‐bonding interactions between the hydroxyl groups of alginates and the phenolic/terpene compounds of the essential oil. Similarly, Iqbal et al. (2023) reported that sodium alginate films containing oregano essential oil exhibited changes in the —OH and carboxylate bands due to hydrogen bond formation. In the present study, such changes were also detected in the 3200–3400 cm^−1^ region. Moreover, shifts in the 1600–1700 cm^−1^ region indicated the presence of carbonyl and aldehyde groups of citral. Lopes et al. (2025) also noted the appearance of C—H and C=O bands of the essential oil within the alginate spectrum, suggesting overlapping interactions between the two compounds. In the current study, the changes in the 2800–3000 cm^−1^ region were attributed to the C—H groups of monoterpenes, while variations in the 1000–1100 cm^−1^ bands were associated with interactions between the C—O—C groups of the polysaccharide and essential oil compounds. Furthermore, with increasing essential oil concentration (from 0.25% to 0.5% and 1%), the intensity of some bands, particularly in the —OH and C—H regions, shifted and became more pronounced. In the 1% essential oil sample, these changes were most evident, indicating the efficient incorporation of LVEOs into the sodium alginate matrix, which may enhance the functional properties of the coating, particularly its antioxidant and antimicrobial activities.

## 4 Chemical Changes

### 4.1 pH

**FIGURE 4:**
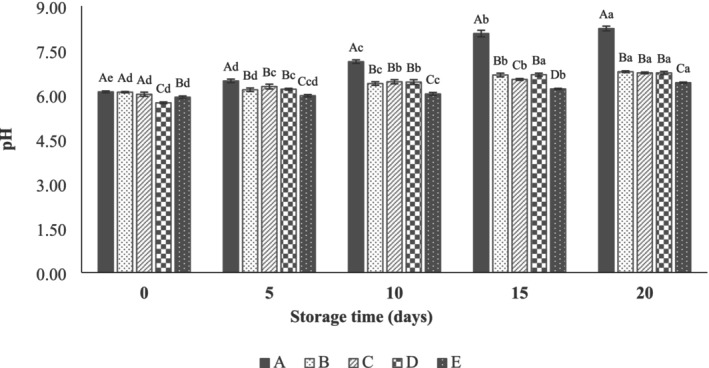
Effect of sodium alginate nanoparticles (SANPs) containing *lemon verbena* plant leaf essential oil (LVEOs) on pH of beef thigh at 4°C for 20 days storage; Treatments = A: Control, B: Sample coated with sodium alginate coating (SA), C: Sample coated with sodium alginate coating and 0.25% *L. verbena* EOs (SA + 0.25% LVEOs), D: Sample coated with SANPs coating containing 0.5% *L. verbena* EOs (SANPs + 0.5% LVEOs), and E: Sample coated with SANPs coating containing 1% *L. verbena* EOs (SANPs + 1% LVEOs). Different capital and small letters indicate statistical differences among times and treatments, respectively (*p* < 0.05).

The initial pH value of the beef thigh samples was ~6.0 (Figure 4), which was similar to the amount reported for beef meat by Alirezalu et al. (2021). According to the results, the pH values of all groups increased during storage (*p* < 0.05). However, this increase was significantly lower in coated samples (*p* < 0.05). Furthermore, using SANPs with LVEOs at higher EO concentration (1%) was more effective, and the pH of samples coated with SANPs+1% LVEO was 6.41 ± 0.02 at the end of the storage. This is due to the lower size and higher ZP.

A smaller particle size and PDI, coupled with a higher zeta potential, enhance the penetration of the EOs into the microorganism's membrane. Moreover, the mentioned factors facilitate the release of active compounds, resulting in antimicrobial and antioxidant properties of this coating in higher concentrations (1%) compared to 0.5% and 0.25%, which can reduce microbial growth and prevent the decomposition of proteins and other nitrogenous compounds, such as triethylamine and ammonia (Shahidi and Hossain 2022). Ghasemi et al. (2024) reported similar results for beef meat coated with carboxymethyl cellulose‐Okra mucilage‐ZnO nanoparticles‐savory EOS. In another research, Cheng et al. (2021) reported pH values of 7.0 in beef slice meat coated with chitosan containing ϵ‐polylysine and glutathione.

### 4.2 TBARS Value

**FIGURE 5:**
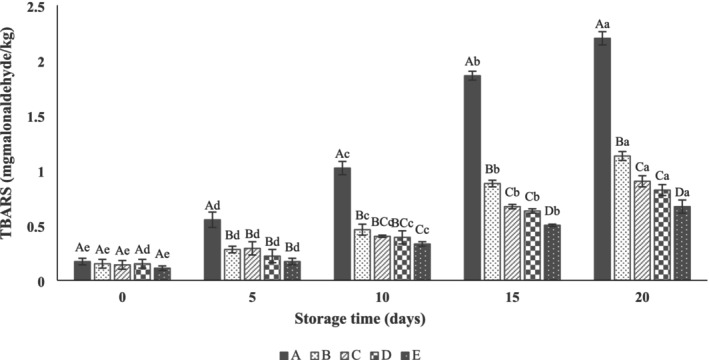
Effect of sodium alginate nanoparticles (SANPs) containing *lemon verbena* plant leaf essential oil (LVEOs) on lipid oxidation (Thiobarbituric acid reactive substances [TBARS]) values of beef thigh at 4°C for 20 days storage; Treatments = A: Control, B: Sample coated with sodium alginate coating (SA), C: Sample coated with sodium alginate coating and 0.25% *L. verbena* EOs (SA + 0.25% LVEOs), D: Sample coated with SANPs coating containing 0.5% *L. verbena* EOs (SANPs + 0.5% LVEOs), and E: Sample coated with SANPs coating containing 1% *L. verbena* EOs (SANPs + 1% LVEOs). Different capital and small letters indicate statistical differences among times and treatments, respectively (*p* < 0.05).

Malondialdehyde (MDA), a dialdehyde, is a secondary product of lipid rancidity, as measured by the TBARS assay (Al‐Dalali et al. 2022). The maximum acceptable TBARS value for fresh meat is 1 mg MDA/kg, beyond which lipid oxidation and chemical spoilage are significant (Hastaoğlu et al. 2021). According to the results (Figure 5), the initial TBARS value was between 0.11 and 0.17 mg MDA/kg sample, which increased gradually during the storage (*p* < 0.05). Moreover, there were significant differences between the control and treatment groups (*p* < 0.05). As can be seen, the TBARS value of the control (1.02 ± 0.06 mg MDA/kg) exceeded the limit range on the 10th day, while it was after the 20th day for the SA‐coated samples (1.13 ± 0.04 mg MDA/kg). Also, the TBARS value of the SANPs +1% LVEOs‐coated group reached 0.67 ± 0.06 mg MDA/kg on Day 20, indicating effective protection against lipid oxidation due to the lower size, homogenous dispersity, and higher ZP, which cause sustained release into thigh beef over time and retard lipid oxidation. Previous studies have shown that the TBARS values of beef meat samples packed were remarkably lower than those of unpacked samples, which is related to their protective effect against oxygen diffusion and reduction of lipid oxidation (Elhadef et al. 2024; Ghasemi et al. 2024; Sayadi et al. 2022). This study demonstrated that adding SANPs +1% LVEOs (containing all compounds) effectively reduced the TBARS value by 69.54% compared with the control, highlighting the strong antioxidative effect of the LVEO‐enriched nanocoating.

### 4.3 TVB‐N Value

**FIGURE 6:**
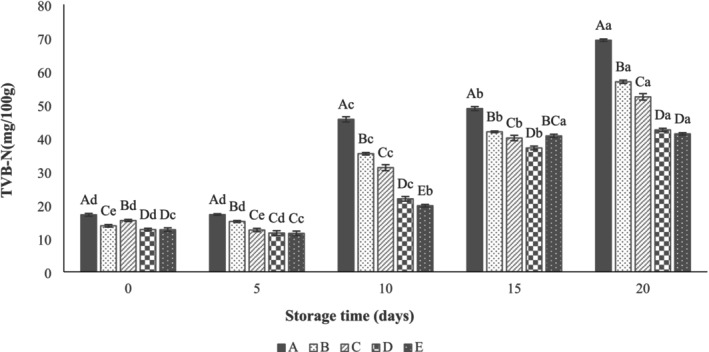
Effect of sodium alginate nanoparticles (SANPs) containing *lemon verbena* plant leaf essential oil (LVEOs) on total volatile basic nitrogen (TVB‐N) values of beef thigh at 4°C for 20 days of storage; Treatments = A: Control, B: Sample coated with sodium alginate coating (SA), C: Sample coated with sodium alginate coating and 0.25% *L. verbena* EOs (SA + 0.25% LVEOs), D: Sample coated with SANPs coating containing 0.5% *L. verbena* EOs (SANPs + 0.5% LVEOs), and E: Sample coated with SANPs coating containing 1% *L. verbena* EOs (SANPs + 1% LVEOs). Different capital and small letters indicate statistical differences among times and treatments, respectively (*p* < 0.05).

Dimethylamine, triethylamine, NH_3_, and other volatile nitrogenous compounds are TVN indices associated with the freshness of meat and meat products. The maximum acceptable limit for TVB‐N in beef meat is 25 mg/100 g, and values above this threshold indicate the onset of spoilage (Bekhit et al. 2021). The TVB‐N values of all beef thigh samples are illustrated in Figure 6. According to the results, the coating application significantly inhibited the increase in TVB‐N levels, and there were significant differences between control and coated samples (*p* < 0.05). As can be seen, the TVB‐N of uncoated beef thigh increased from 17.06 ± 0.41 to 69.29 ± 0.36 mg/100 g over 20 days of refrigerated storage, confirming spoilage at the end of storage. In contrast, the values were 42.46 ± 0.49 and 41.28 ± 0.34 mg N/100 g, respectively, for those treated with nanocoatings loaded with 0.5% and 1% LVEOs. Comparison of the groups showed that the TVB‐N value of the control on Day 10 of storage (45.63 ± 0.79 mg of N/100 g) exceeded the maximum acceptable range, whereas the TVB‐N value of the SANPs + 1% LVEOs (19.78 ± 0.40 mg of N/100 g) was within the acceptable range. This can be attributed to a reduction in the growth of spoilage bacteria, which aligns with the microbial results (Hojati et al. 2024; Rezaeifar et al. 2020). Similarly, Li et al. (2021) observed that the addition of 0.3% LVEOs to active coating decreased the TVB‐N values from 41.77 to 25.26 mg N/100 g in a large yellow croaker after 15 days of storage at 4°C. In another study, Osanloo et al. (2023) reported lower TVB‐N content in shrimp samples coated with SANPs that incorporated *Cuminum cyminum* and Zataria multiflora. At the end of the storage period, the addition of SANPs + 1% LVEOs (containing all compounds) reduced the TVB‐N value to 40.42%.

### 4.4 Microbial Changes

**FIGURE 7:**
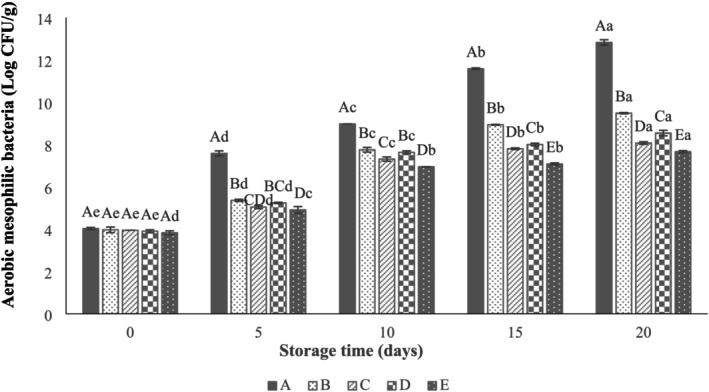
Effect of sodium alginate nanoparticles (SANPs) containing *lemon verbena* plant leaf essential oil (LVEOs) on aerobic mesophilic bacteria in coated and uncoated beef thigh stored at 4°C for 20 days; Treatments = A: Control, B: Sample coated with sodium alginate coating (SA), C: Sample coated with sodium alginate coating and 0.25% *L. verbena* EOs (SA + 0.25% LVEOs), D: Sample coated with SANPs coating containing 0.5% *L. verbena* EOs (SANPs + 0.5% LVEOs), and E: Sample coated with SANPs coating containing 1% *L. verbena* EOs (SANPs + 1% LVEOs). Different capital and small letters indicate statistical differences among times and treatments, respectively (*p* < 0.05).

**FIGURE 8:**
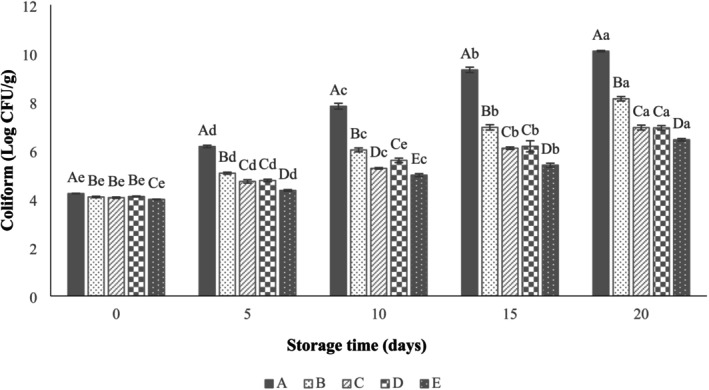
Effect of sodium alginate nanoparticles (SANPs) containing *lemon verbena* plant leaf essential oil (LVEOs) on Coliform Count in coated and uncoated beef thigh stored at 4°C for 20 days; Treatments = A: Control, B: Sample coated with sodium alginate coating (SA), C: Sample coated with sodium alginate coating and 0.25% *L. verbena* EOs (SA + 0.25% LVEOs), D: Sample coated with SANPs coating containing 0.5% *L. verbena* EOs (SANPs + 0.5% LVEOs), and E: Sample coated with SANPs coating containing 1% *L. verbena* EOs (SANPs + 1% LVEOs). Different capital and small letters indicate statistical differences among times and treatments, respectively (*p* < 0.05).

**FIGURE 9:**
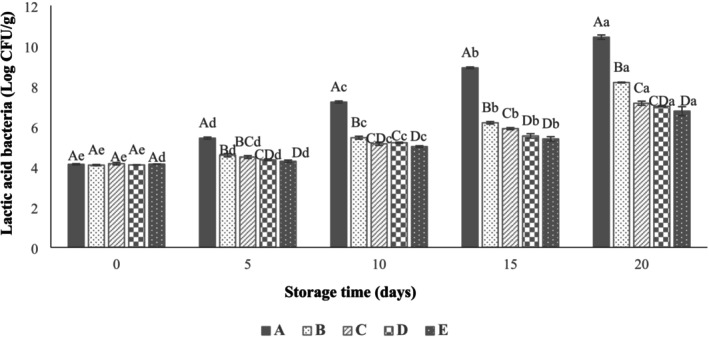
Effect of sodium alginate nanoparticles (SANPs) containing *lemon verbena* plant leaf essential oil (LVEOs) on Lactic Acid Bacteria (LAB) Count in coated and uncoated beef thigh stored at 4°C for 20 days; Treatments = A: Control, B: Sample coated with sodium alginate coating (SA), C: Sample coated with sodium alginate coating and 0.25% *L. verbena* EOs (SA + 0.25% LVEOs), D: Sample coated with SANPs coating containing 0.5% *L. verbena* EOs (SANPs + 0.5% LVEOs), and E: Sample coated with SANPs coating containing 1% *L. verbena* EOs (SANPs + 1% LVEOs). Different capital and small letters indicate statistical differences among times and treatments, respectively (*p* < 0.05).

Based on ICMSF (2018), the acceptable limits for raw beef are ≤ 7 log CFU/g for aerobic mesophilic bacteria and LAB, and ≤ 2–3 log CFU/g for coliforms (ICMSF 2018). The microbial quality of different beef thighs during refrigerated storage is shown in Figures 7, 8, 9. According to Figure 7, the initial aerobic mesophilic bacteria count of beef thigh was approximately 4 log CFU/g and increased significantly during storage (*p* < 0.05). Moreover, incorporation of LVEOs (1%) significantly impacts the aerobic mesophilic bacteria count (*p* < 0.05), followed by LVEOs (0.5%) and LVEOs (0.25%). According to the results, the aerobic mesophilic bacteria counts of control, SA, SANPs +0.25% LVEOs, SANPs +0.5% LVEOs, and SANPs+1% LVEOs groups reached 12.82 ± 0.18, 9.48, 8.07 ± 0.04, 8.54 ± 0.18, and 7.66 ± 0.06 log CFU/g at the end of the storage (Figure 7). Zhang et al. (2021) found that total viable counts were decreased in beef meat coated in sodium alginate–agar film containing ginger EOs. The shelf life increased to 11 days at 4°C. In the study conducted by Sayadi Sayadi, Mojaddar Langroodi, and Jafarpour (2021), stated a similar effect of *Pimpinella anisum* EO and ginger extract added to zein coating solution on reducing aerobic mesophilic bacteria counts of bovine meat.

According to Figure 8, the coliform count of beef thighs uncoated was around 4.22 ± 0.01 log CFU/g on the start day of storage and then increased (10.09 ± 0.04 log CFU/g) during refrigeration (20 days of storage). Compared to the other treated samples, the SANPs +1% LVEOs‐coated group had a significantly lower coliform count, indicating a better antimicrobial effect of nanocoatings with EOs. After 20 days, SA, SANPs +0.25% LVEOs, SANPs +0.5% LVEOs, and SANPs +1% LVEOs films displayed coliform counts equal to 8.13 ± 0.12, 6.94 ± 0.13, 6.93 ± 0.12, and 6.44 ± 0.07 log CFU/g, respectively. In a study by Behbahani et al. (2020), the inhibitory effect of Shahri balanced seed mucilage coating incorporated with cumin EOs on the growth of coliform bacteria in beef samples was reported. Similarly, Sarmadikia et al. (2022) stated a decline in coliform growth when bitter orange peel extract was added to the quince seed mucilage coating matrix.

In addition, the initial counts of LAB (Figure 9) were between 4.08–4.15 log CFU/g in all samples. At the end of storage, the counts of LAB reached 10.43 ± 0.15 log CFU/g for the uncoated group. Moreover, coating with SANPs containing LVEOs at two levels (0.5% and 1%) in the coating of beef thigh samples decreased LAB to 7.00 ± 0.04 and 6.76 ± 0.30 log CFU/g at the end of storage. (Sani et al. 2017) imparted the inhibitory impact of whey protein isolate/cellulose nanofiber films containing TiO_2_ NPs and rosemary EOs on the LAB growth in lamb meat could reduce the LAB count. It was also represented that packing turkey meat with a chitosan coating combined with *Origanum vulgare* EO and grape seed extract could reduce the LAB count (Mojaddar Langroodi et al. 2021). In this study, microbial counts of the control group clearly exceeded these limits, confirming spoilage. Coated samples, especially those containing SANPs and 1% LVEOs, showed significantly reduced growth, keeping LAB counts close to the acceptable threshold, although coliform counts were still higher than the standard. These results indicate that coatings enriched with LVEOs can effectively delay microbial growth and extend shelf life; however, further interventions may be necessary to fully meet safety standards.

### 4.5 Sensory Evaluation

**FIGURE 10:**
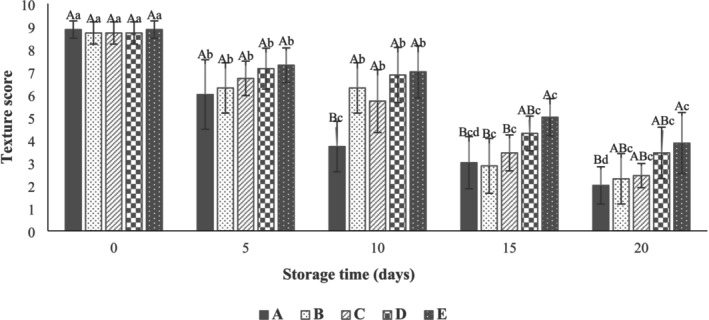
Texture scores of beef thigh during storage at 4°C for 20 days; Treatments = A: Control, B: Sample coated with sodium alginate coating (SA), C: Sample coated with sodium alginate coating and 0.25% *L. verbena* EOs (SA + 0.25% LVEOs), D: Sample coated with SANPs coating containing 0.5% *L. verbena* EOs (SANPs + 0.5% LVEOs), and E: Sample coated with SANPs coating containing 1% *L. verbena* EOs (SANPs + 1% LVEOs). Different capital and small letters indicate statistical differences among times and treatments, respectively (*p* < 0.05).

**FIGURE 11:**
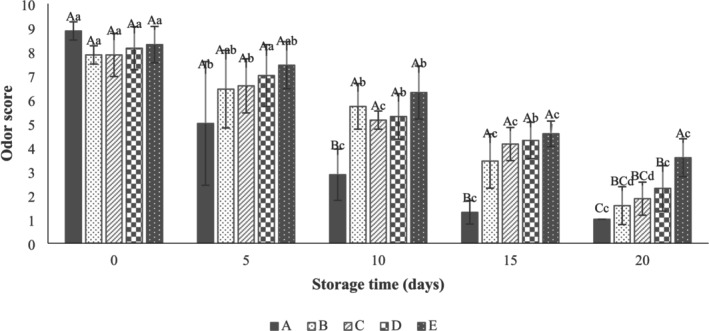
Odor scores of beef thigh during storage at 4°C for 20 days; Treatments A: Control, B: Sample coated with sodium alginate coating (SA), C: Sample coated with sodium alginate coating and 0.25% *L. verbena* EOs (SA + 0.25% LVEOs), D: Sample coated with SANPs coating containing 0.5% *L. verbena* EOs (SANPs + 0.5% LVEOs), and E: Sample coated with SANPs coating containing 1% *L. verbena* EOs (SANPs + 1% LVEOs). Different capital and small letters indicate statistical differences among times and treatments, respectively (*p* < 0.05).

**FIGURE 12:**
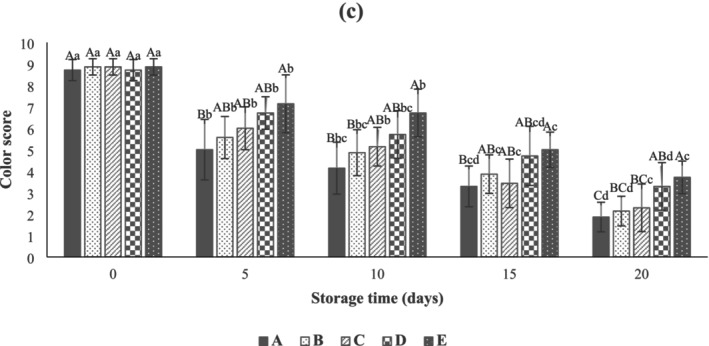
Color scores of beef thigh during storage at 4°C for 20 days; Treatments A: Control, B: Sample coated with sodium alginate coating (SA), C: Sample coated with sodium alginate coating and 0.25% *L. verbena* EOs (SA + 0.25% LVEOs), D: Sample coated with SANPs coating containing 0.5% *L. verbena* EOs (SANPs + 0.5% LVEOs), and E: Sample coated with SANPs coating containing 1% *L. verbena* EOs (SANPs + 1% LVEOs). Different capital and small letters indicate statistical differences among times and treatments, respectively (*p* < 0.05).

**FIGURE 13:**
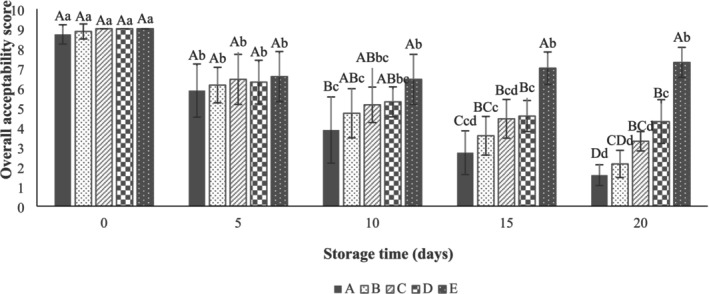
Overall acceptability scores of beef thigh during storage at 4°C for 20 days; Treatments A: Control, B: Sample coated with sodium alginate coating (SA), C: Sample coated with sodium alginate coating and 0.25% *L. verbena* EOs (SA + 0.25% LVEOs), D: Sample coated with SANPs coating containing 0.5% *L. verbena* EOs (SANPs + 0.5% LVEOs), and E: Sample coated with SANPs coating containing 1% *L. verbena* EOs (SANPs + 1% LVEOs). Different capital and small letters indicate statistical differences among times and treatments, respectively (*p* < 0.05).

The results showed that there was no significant difference (*p* > 0.05) between the sensory properties (texture, color, odor, and overall acceptability) of beef thigh samples (Figures 10, 11, 12, 13) in all treatments at Day 0. As can be seen in Figure 10, the control samples (3.71 ± 1.11) after 10 days of storage, the SA‐coated samples (2.86 ± 1.21), and the SA + 0.25% LVEOs (3.43 ± 0.79) after 15 days of storage were unacceptable in terms of texture, respectively. The beef thigh samples nanocoated with 0.5% (4.29 ± 0.76) and 1% (3.86 ± 1.35) LVEOs were acceptable after 15 and 20 days of storage, respectively. Moreover, the odor scores of samples had the same trend, with the control and all treated samples being unacceptable after 10 and 15 days of storage, respectively (Figure 11). For the color change, the control (4.14 ± 1.21), SA (4.86 ± 1.07) after 10 days, SA + 0.25% LVEOs (3.43 ± 1.13), SANPs + 0.5% LVEOs (4.71 ± 1.38) after 15 days, and SANPs + 1% LVEOs (3.71 ± 0.76) samples became unacceptable after 20 days of storage, respectively (Figure 12). The levels of 1% LVEOs added to nanocoating at the end of the storage had high acceptability (7.29 ± 0.76) (Figure 13). Similar results have been stated in former studies of chitosan coating incorporated with EO and the extract of *lemon verbena* for rainbow trout (Rezaeifar et al. 2020), gelatine film containing nano TiO_2_ and cumin EO on fresh chicken (Sayadi, Amiri, and Radi 2021), and sodium alginate–agar coating containing ginger EO on beef (Zhang et al. 2021).

## 5 Conclusion

The current study indicated that coating beef thighs with SANPs containing *lemon verbena* EOs was greatly efficient against spoilage bacteria such as aerobic mesophilic, coliform, and LAB. The nanocoatings also retarded lipid oxidation and exhibited acceptable sensory properties, thereby extending the shelf life of fresh beef thighs from 5 to 15 days at 4°C. Additionally, beef thighs treated with all the compounds (SANPs + 1% LVEOs) showed the highest microbiological, chemical, and sensory quality. Therefore, SA nanoparticles combined with lemon verbena EOs could be a useful novel active packaging for protecting fresh meat under refrigerated storage.

## Author Contributions

**Mehran Sayadi:** conceptualization (equal), data curation (equal), data curation (equal), methodology (equal), methodology (equal), project administration (equal), project administration (equal). **Elahe Abedi:** project administration (equal), writing – original draft (equal), writing – review and editing (equal). **Zolaikha Shiravani:** software (equal), writing – original draft (equal). **Niayesh Ramyar:** data curation (equal), formal analysis (equal), resources (equal). **Issa Mohammadpourfard:** investigation (equal), supervision (equal), supervision (equal), validation (equal), validation (equal). **Seyed Mohammad Bagher Hashemi:** project administration (equal), writing – review and editing (equal). **Zahra Eskandari:** methodology (equal), project administration (equal). **Narjes Jamali:** validation (equal).

## Ethics Statement

This article does not contain any studies with human participants or animals performed by any of the authors.

## Conflicts of Interest

The authors declare no conflicts of interest.

## Data Availability

Research data are not shared.
